# Parametric study of EEG sensitivity to phase noise during face processing

**DOI:** 10.1186/1471-2202-9-98

**Published:** 2008-10-03

**Authors:** Guillaume A Rousselet, Cyril R Pernet, Patrick J Bennett, Allison B Sekuler

**Affiliations:** 1 Centre for Cognitive Neuroimaging (CCNi) and Department of Psychology, University of Glasgow, Glasgow, UK; 2 SFC Brain Imaging Research Centre, Division of Clinical Neurosciences, Western General Hospital, Edinburgh, UK; 3 McMaster University, Department of Psychology, Behaviour & Neuroscience, Hamilton, ON, Canada

## Abstract

**Background:**

The present paper examines the visual processing speed of complex objects, here faces, by mapping the relationship between object physical properties and single-trial brain responses. Measuring visual processing speed is challenging because uncontrolled physical differences that co-vary with object categories might affect brain measurements, thus biasing our speed estimates. Recently, we demonstrated that early event-related potential (ERP) differences between faces and objects are preserved even when images differ only in phase information, and amplitude spectra are equated across image categories. Here, we use a parametric design to study how early ERP to faces are shaped by phase information. Subjects performed a two-alternative force choice discrimination between two faces (Experiment 1) or textures (two control experiments). All stimuli had the same amplitude spectrum and were presented at 11 phase noise levels, varying from 0% to 100% in 10% increments, using a linear phase interpolation technique. Single-trial ERP data from each subject were analysed using a multiple linear regression model.

**Results:**

Our results show that sensitivity to phase noise in faces emerges progressively in a short time window between the P1 and the N170 ERP visual components. The sensitivity to phase noise starts at about 120–130 ms after stimulus onset and continues for another 25–40 ms. This result was robust both within and across subjects. A control experiment using pink noise textures, which had the same second-order statistics as the faces used in Experiment 1, demonstrated that the sensitivity to phase noise observed for faces cannot be explained by the presence of global image structure alone. A second control experiment used wavelet textures that were matched to the face stimuli in terms of second- and higher-order image statistics. Results from this experiment suggest that higher-order statistics of faces are necessary but not sufficient to obtain the sensitivity to phase noise function observed in response to faces.

**Conclusion:**

Our results constitute the first quantitative assessment of the time course of phase information processing by the human visual brain. We interpret our results in a framework that focuses on image statistics and single-trial analyses.

## Background

In primates, visual object processing unfolds from the retina to higher-order cortical areas through a hierarchy of processing steps. Although, at the neuronal level, lateral and feedback connections are integrated to the feedforward sweep of information [1,2], at the functional level, neuronal mechanisms can still be conceptualized as performing rapid transformations of the input retinal activation to achieve increasingly refined representations [3]. A fundamental question in vision science is thus how to uncover the mechanisms by which the pattern of retinal activation is progressively transformed into a code that is useful for making behavioural decisions. In recent years there has been an on-going debate as to what stimuli are best for probing visual neuronal mechanisms. This debate stems mostly from the study of neurons in V1, the primary visual cortex, and whether their visual response properties can be better understood by using simple well-controlled stimuli [4], or natural scene stimuli, the type of stimuli the visual system might have evolved to apprehend best [5]. At the other end of the visual cortical hierarchy, in higher order visual areas, no such debate exists since those areas are mostly responsive to complex objects, and not to simple patterns [6-10]. In those areas, the emphasis has been put on object categories and their relative specificity [11]. Although interesting in itself, the category-related parcelling of the visual cortex ignores the question of the transformation mechanisms taking place along the visual hierarchy. Because these processes occur very fast, critical information processing events may be observed at the time-scale of EEG (electroencephalography) [3,12-14]. In humans, EEG (as well as MEG more recently) has revealed a cascade of neuronal activations following stimulus presentation [15]. Within 200 ms, neuronal activity has been reported that dissociates among various object categories, in particular faces and words [16-19]. In particular, the larger ERP component to faces and words, compared to other control categories, and peaking at about 170 ms, the N170 [20-23], has been the subject of much debate about its categorical sensitivity [24]. Early activity, in the time window of the P1 component (80–120 ms), also has been discussed as a potential marker of complex object processing [25-27].

On-going controversies about the time-course of object processing are due, in part, to the difficulty associated with controlling the effects of low-level sensory variables on higher-order perceptual operations. In classic categorical designs that are used to assess object-processing speed, uncontrolled physical properties tend to co-vary with the object categories that are contrasted. Such physical properties might introduce biases in our brain measurements that are unrelated to the higher-level object processing that are meant to be measured, but instead reflect the extraction of visual information by lower levels of the visual hierarchy [25,28].

Recent advances have revealed that the activity in the N170 time window, but not earlier activity, is related to the extraction of task-related information (EEG: [14,29-31]; MEG: [32,33]). These advances were made possible by a tight control of the stimulus space that relied on parametric, rather than categorical, designs. Parametric designs are well suited to explore brain dynamics in a systematic fashion because, by varying one or several parameters along a continuum, they can provide a genuine information space and stronger constraints on statistical analyses [14,34,35].

**Figure 1:**
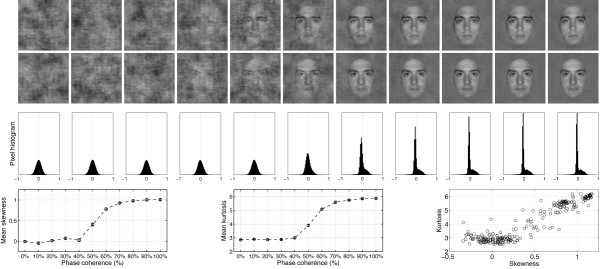
**Examples of stimuli used in Experiment 1**. The first two rows show the 22 stimuli presented to one observer during the first block of the experiment. The observer discriminated the same two faces during the whole experiment. The noise level varied from 100% (left side; 0% phase coherence) to 0% (right side; 100% phase coherence). Note that at each level of phase coherence the structure of the noise that was mixed with the original image was different, so that the task could not be performed based on the spatial characteristics of the noise. Histograms in the third row show the distribution of pixel contrasts averaged across all stimuli seen by this observer at each level of phase coherence. Starting with a Gaussian distribution (left), the pixel histograms become increasingly skewed and kurtotic with increasing phase coherence (right – the y-axes on the histograms are all the same). This relationship is depicted in the last row, showing the mean skewness (left), and mean kurtosis (middle), as a function of phase coherence. The error bars correspond to 95% confidence interval computed using a bootstrap percentile technique (1000 resamples). In the bottom right end graph, kurtosis for each image (each circle) is expressed as a function of skewness. Although the two statistical descriptors are correlated, the relationship is not linear. As demonstrated below, EEG amplitude is more sensitive to the kurtosis of the image than its skewness.

The goal of our project was to build on this new literature using parametric designs, to determine what image properties drive early responses to objects. Images are characterized by their amplitude and phase spectra in the Fourier domain. Differences in amplitude spectra are not likely to explain early responses to faces and objects because equating amplitude spectra across stimuli preserve those differences [28,30,31,36,37]. For instance, in Figure 1, the top left textures and the top right faces all have the same amplitude spectrum, but have different phase spectra. Therefore, phase information is the most likely candidate, a conclusion that has been reached previously for psychophysical data [38-41]. When contrasting face stimuli to noise textures created by complete randomization of the phase information while keeping the amplitude information constant, the earliest ERP differences occur at about 120–140 ms after stimulus onset [28,30,31,37,42,43]. Although this might be the time at which object-related global phase information is extracted by the visual system, the precise time course of this process is still unknown, as is the way phase information influences early cortical responses to objects. We addressed these questions by manipulating phase information systematically along a continuum while subjects discriminated between two faces briefly presented on a screen (Figure 1). Mutual phase information, with respect to the target images, was manipulated by adding phase noise in linear steps. Analyses were performed on each subject at the single-trial level, using a linear regression model that contained only predictors related to physical image characteristics, including stimulus type (e.g., face 1 or face 2) and percentage of phase information (ranging from 100%, i.e. original stimuli, to 0%, i.e. equivalent to the phase scrambled textures used in earlier experiments).

Previous experiments have shown that behavioural performance of human observers in natural scene categorisation tasks is very resistant to linear alterations of the phase spectrum by introduction of phase noise [40,44,45]. This finding might be explained by the existence of non-linearities in higher-order image statistics introduced by linear manipulations of phase information [46]. In particular, phase manipulations affect the skewness and kurtosis of natural images (Figure 1), i.e. their 3^rd ^and 4^th ^order statistics. Skewness is a measure of the asymmetry of a distribution. It equals zero for a Gaussian distribution, and is negative or positive for distributions with more variance to the left or to the right of their centre. Kurtosis is a measure of how peaky and heavy-tailed a distribution is, and equals 3 for a Gaussian distribution [47]. An image with a high kurtosis contains a large proportion of almost identical pixels, and few pixels that differ significantly from the modal value. Skewness and kurtosis are defined as the normalized 3^rd ^and 4^th ^moments about the mean of the pixel luminance distribution [48,49]:

$$\text{Skewness}=\frac{1}{N}\left(\sum_{i}^{N}(x_{i}-\bar{x}{)}^{3}\right)\frac{1}{{\left(\sigma^{2}\right)}^{3/2}};\text{Kurtosis}=\frac{1}{N}\left(\sum_{i}^{N}(x_{i}-\bar{x}{)}^{4}\right)\frac{1}{{\left(\sigma^{2}\right)}^{2}}$$

where N is the sample size of the distribution, x_i _the value of the *i*-th member of the distribution, $\bar{\text{x}}$ the mean of the *i *values, and *σ*^2 ^the variance (for a good visualisation of skewness and kurtosis in the context of 1/f wavelet textures, see Figure 1 from [48]). Kurtosis, in particular, seems to be related to the presence of edges and local contours in natural images, and might thus be a better indicator of image structure than global phase coherence *per se *[46,50-52]. Therefore, we included skewness and kurtosis as predictors in our linear regression model. We report data showing, in response to face stimuli, sensitivity to phase noise that emerged very rapidly at the transition between the P1 and the N170 components, in the 120–150 ms after stimulus onset time window.

## Methods

### Participants

A total of 10 subjects participated in one main experiment and two control experiments. Experiment 1 included eight subjects (five males and three females). Four were tested twice (test-retest, on two different days), leading to a total of 12 experimental sessions. Only half of the subjects were tested twice because of the robust replication of the effects we obtained with the first four subjects (see Results). Subjects' mean age was 24 years old (min = 21, max = 28, SD = 2.5); seven were right handed. Experiment 2 included four male subjects, and all of them were tested twice (mean age 27, min = 24, max = 29, SD = 2.4; three right handed). Four subjects (one female) participated in Experiment 3, one of whom was tested twice (mean age 25, min = 22, max = 29, SD = 3; three were right handed). All subjects gave written informed consent and had normal or corrected-to-normal vision. Five subjects participated in one experiment only, four participated in two experiments, and only one subject participated in three experiments. This last subject, RXL, was singled-out for this reason in the results. Among the 10 different individuals, 4 received $10/hour for their participation; the others were members of the laboratory and were not compensated for participation. The McMaster University Research Ethics Board approved the research protocol.

### Stimuli

One pair of female faces and one pair of male faces were selected from a set of 10 faces used in previous experiments [36,53,54]. Each subject saw only two faces, from the same gender, and male and female faces were counterbalanced across subjects. These faces were front-view greyscale photographs cropped within a common oval frame and pasted on a uniform 10° × 10° background (Figure 1). Face stimuli all had the same mean amplitude spectrum and thus differed only in terms of phase information, which carries most of the form information [55,56]. We created four noise textures by randomizing the phase of the four faces. Thus, these patterns, which we refer to as pink noise textures, had the same amplitude spectrum as the faces, but differed from faces in terms of higher-order statistics. We also created four wavelet textures, each matched for the global image statistics of one of the faces. These textures not only matched the skewness and kurtosis of the original face stimuli, they also matched properties such as local and long-distance multiscale phase correlations. The textures were created with the Matlab toolbox provided by Portilla and Simoncelli ([57], http://www.cns.nyu.edu/~lcv/texture/), with the parameters set to four scales, four orientations, a 9 × 9 spatial neighbourhood, and 50 iterations.

Phase spectra were manipulated using the weighted mean phase technique (WMP, [46]), so that images were characterized by their percentage of phase coherence. Starting from the original phase of an image *φ*_*image*_, the final phase *φ*_*final *_was computed by the following equation:

$$\begin{array}{cc} \varphi_{final}=\left\{ \begin{array}{l} \tan^{-1}(S_{\varphi }/C_{\varphi })\\ \tan^{-1}(S_{\varphi }/C_{\varphi })+\pi \\ \tan^{-1}(S_{\varphi }/C_{\varphi })+2\pi \end{array}\right. & \begin{array}{l} C_{\varphi }>0\\ C_{\varphi }<0,S_{\varphi }>0\\ C_{\varphi }<0,S_{\varphi }<0 \end{array}\\ \begin{array}{l} where:\\ S_{\varphi }=w\sin(\varphi_{image})+(1-w)\sin(\varphi_{noise})\\ C_{\varphi }=w\cos(\varphi_{image})+(1-w)\cos(\varphi_{noise}) \end{array} & w[0,1] \end{array}$$

This technique takes into account the directional nature of phase, assuring that phases are uniformly distributed after transformation. In comparison, a strict linear blend would lead to an over-representation of phases around 0°. Thus, WMP has the advantage over a linear blend technique to produce monotonic changes in third-order (skewness) and fourth-order (kurtosis) image statistics, as illustrated at the bottom part of Figure 1 and in [46]. Kurtosis is often used as a measure of image sparseness and is highly correlated with the representation of phase structure, high levels of kurtosis corresponding to local phase-congruent structures such as edges [50].

For all stimuli, pixel contrasts ranged between -1 and 1, with a mean of 0. RMS contrast was kept constant across all levels of phase coherence.

### Experimental design

**Figure 2:**
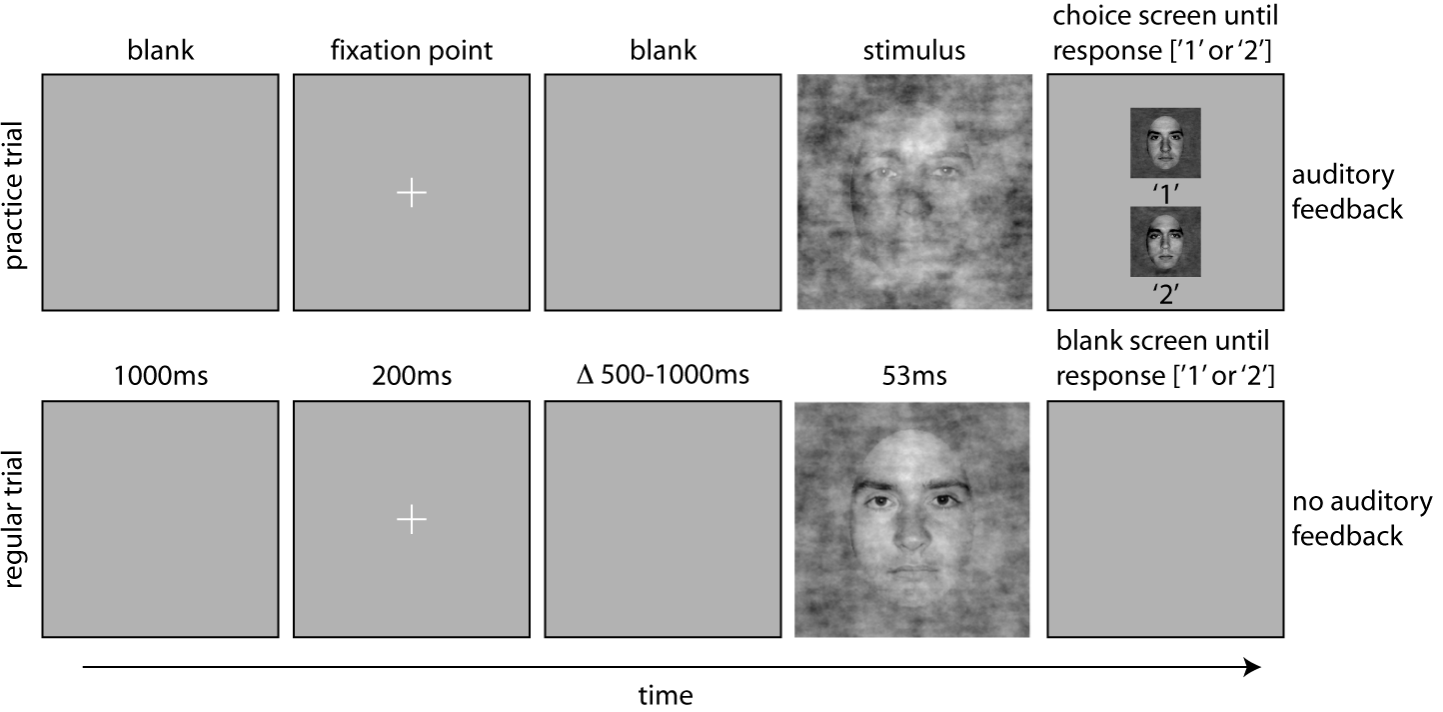
**Organisation of practice trials (top row) and regular experimental trials (bottom row) in all experiments**. The stimuli are examples taken from the main experiment that used faces (Experiment 1). A trial started with a blank screen for 1000 ms, followed by the presentation of a fixation point for 200 ms. Then, after a random delay ranging from 500 to 1000 ms, a stimulus was presented for 53 ms. During practice trials, a choice screen appeared immediately after the stimulus, showing the two targets of the experiment and their associated response keys. The screen stayed on until the subject's response, which was followed by auditory feedback, before the trial sequence resumed. During regular trials, a blank screen appeared immediately after the stimulus, and remained on until the subject's response. No feedback was provided during regular trials. Note that stimuli are not drawn to scale.

Subjects sat in a dimly lit sound-attenuated booth. Viewing distance was maintained at 90 cm with a chinrest. Stimuli were presented for about 53 ms (4 frames at 75 Hz) on a Sony Trinitron GDM-F520 monitor (800 × 600 pixels, effective height and width: 40.5 × 30.5 cm). Subjects were given unlimited time to respond by pressing '1' or '2' on the numerical pad of the keyboard to indicate which stimulus had been displayed (Figure 2). In Experiment 1, subjects had to discriminate between two faces; in Experiment 2 between two pink noise textures; and in Experiment 3 between two wavelet noise textures. In all experiments, subjects were told to emphasize response accuracy, not speed. The button/identity association was assigned randomly for each subject. An experiment consisted of 12 blocks of 132 trials (1584 trials in total with 144 trials per level of phase coherence). Within each block, there were six repetitions of each face or texture in 11 phase coherence levels. Each block was preceded by practice trials that allowed subjects to learn the stimulus-key association (10 in Experiment 1, and 20 in the two control experiments). A regular trial was organized as follows: A blank screen was presented for 1000 ms, followed by a small fixation cross (i.e., a 0.3 deg '+' in the middle of the screen) for 200 ms, after which another blank screen was presented for a random duration ranging from 500 to 1000 ms. Then a stimulus was presented for 53 ms, followed by a blank screen that stayed on until subjects provided their response. Practice trials were very similar, except that immediately after the presentation of the stimulus, a choice screen appeared that showed each face or texture simultaneously, one above the other, with the corresponding label below each item. Auditory feedback was provided after the subject pressed a response key, with low- and high-pitch tones indicating incorrect and correct responses. Feedback was provided only during practice trials.

### EEG recording and analysis

EEG data were acquired with a 256-channel Geodesic Sensor Net (Electrical Geodesics Inc., Eugene, Oregon, [58]). Analog signal was digitized at 500 Hz and band-pass filtered between 0.1 Hz and 200 Hz. Impedances were kept below 50 kΩ. Subjects were asked to minimize blinking, head movement, and swallowing. Subjects were then given a description of the task. EEG data were referenced on-line to electrode Cz and re-referenced off-line to an average reference. The signal was then low-pass filtered at 30 Hz and bad channels removed, with no interpolation. The 30 Hz low-pass filter was justified by a previous study in which we showed that the differential activity evoked by faces and objects is contained mostly in a narrow 5–15 Hz band [37]. Baseline correction was performed using the 300 ms of pre-stimulus activity and data epoched in the range -300 ms to 400 ms. Trials with abnormal activities were excluded based on a detection of extreme values, abnormal trends, and abnormal distributions, using EEGLAB functions [59,60]. The threshold for extreme values was ± 100 *μ*V for all channels. An epoch was rejected for abnormal trend if it had a slope larger than 75 *μ*V/epoch and a regression *R*^2 ^larger than 0.3. An epoch was rejected for abnormal distribution when its kurtosis fell outside five standard deviations of the kurtosis distribution for each single electrode or across all electrodes. All remaining trials were included in the analyses, whether they were associated with correct or incorrect behavioural responses.

Using a multiple linear regression approach, the single-trial EEG amplitude in *μ*V was expressed using one model for all three experiments:

*EEG *= *β*_1 _+ *β*_2_*S *+ *β*_3*φ *_+ *β*_4*γ*2 _+ *β*_5*γ*1 _+ *β*_6*φγ*2 _+ *β*_7*φγ*1 _+ *ε*

The fit was performed at each electrode and each time point independently using the glmfit Matlab function, with a normal distribution. Phase (*φ*), skewness (*γ*_1_) and kurtosis (*γ*_2_) were coded as continuous regressors, while the regressor for stimulus identity (e.g., Face A vs. Face B) (*S*) was a categorical factor. Regression coefficients (*β*) are expressed in an arbitrary unit that reflects the strength of the fit (i.e. the influence of the factor on the EEG signal). The terms (*φγ*_2_) and (*φγ*_1_) correspond to, respectively, phase-kurtosis and phase-skewness interactions. The error term is (*ε*).

For each subject, we report the electrode at which the model provided the best fit (i.e., where R^2 ^was largest). In general, R^2 ^was largest in a cluster of posterior electrodes that also exhibited large N170 responses to faces. In some cases, the largest R^2 ^was obtained at an electrode that did not produce the strongest N170 to faces. For each of these cases, however, the time-course and relative strength of the best-fitting regression parameters were virtually identical at both sites (i.e., the one producing the largest N170 to faces and the other that produced the largest R^2^). In addition to the multiple intra-subject analyses, we evaluated the influence of each regressor on the EEG across subjects using semi-partial correlation coefficients.

## Results

### Experiment 1: discrimination between two faces

**Figure 3:**
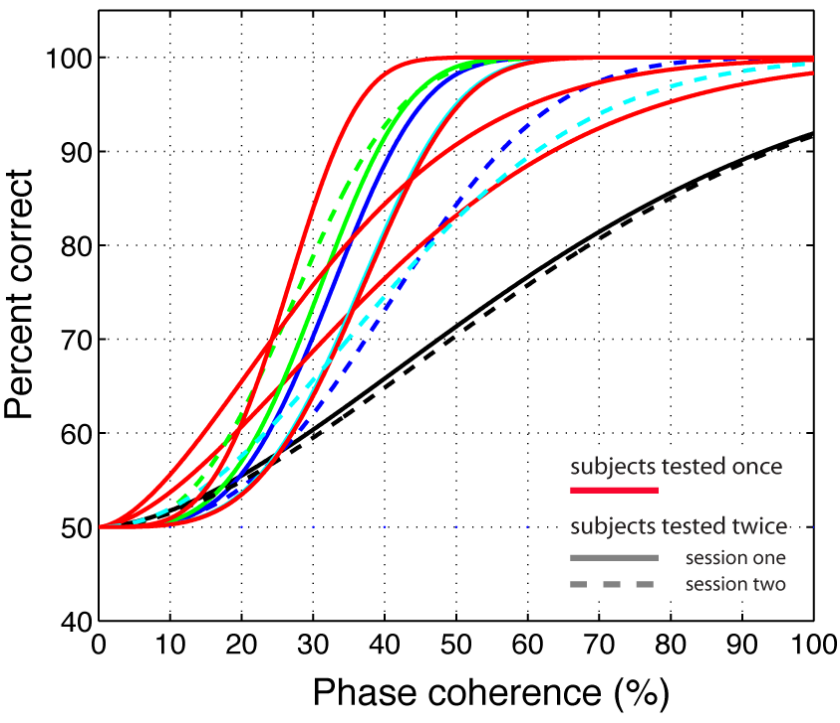
**Percent correct for individual subjects in Experiment 1**. The black lines show data from subject RXL. Subject RXL was singled-out because he is the only subject that was tested in experiment 1 and the two control experiments. The other colours depict data from all other observers and sessions. Continuous lines indicate the first recording session, while dashed lines indicate the second session of subjects who were tested twice. Data from subjects who were tested only once are indicated in red. Data were fit using a cumulative Weibull function where the accuracy *p *was expressed as a function of the phase coherence *c*, the phase coherence *α *supporting 82% threshold performance, and *β *the slope of the curve: $p=1-0.5e^{-{\left(c/\alpha \right)}^{\beta }}$

In terms of RT, subjects presented one of two patterns. Four out of eight subjects showed an inverted U-shape function, with the shortest RT for extreme values of phase coherence, and the longest RT for ambiguous conditions. Among the other four subjects, three showed longer RT at low phase coherence and a sharp transition towards shorter RT at high phase coherence, while one subject showed the opposite pattern. The RT profiles from these last four subjects were correlated with accuracy (first three subjects: r<-.77, p < .01; last subject: r>.76, p < .01). This correlation occurred because response accuracy was near chance levels in all subjects when phase coherence was 0%, and increased significantly with greater phase coherence. Figure 3 shows percent correct for each subject as a function of phase coherence. There was a large variability in response patterns across subjects and sessions but all subjects were at or close to ceiling performance at the highest levels of phase coherence.

**Figure 4:**
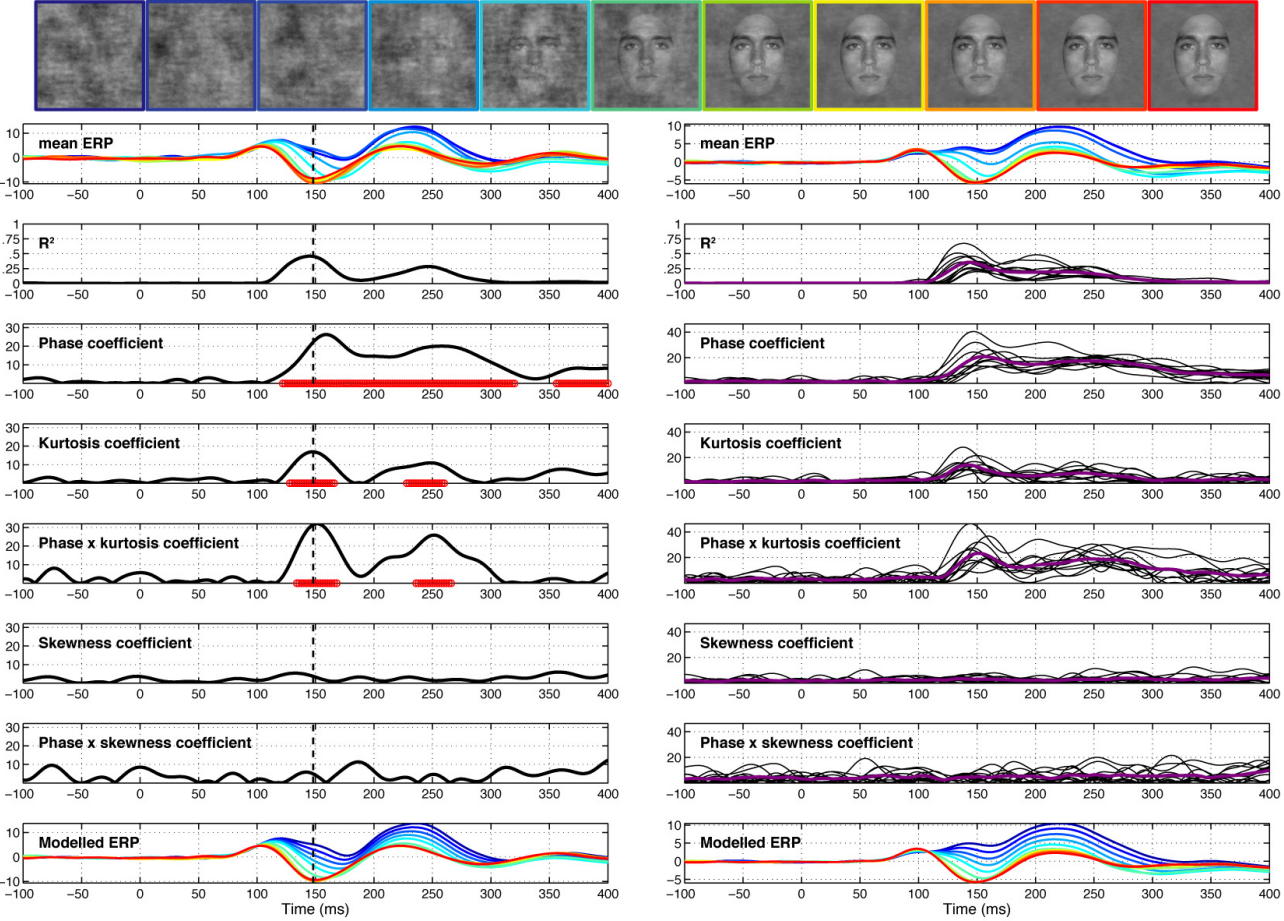
**Session 1 of subject RXL (left) and data averaged across subjects (right) in Experiment 1**. The vertical line in the left column indicates the latency of maximum R^2 ^for the model, recorded at electrode E107 for RXL, an electrode halfway between T5 and O1. Electrode E107 is at the centre of the left red cluster in the topographic map of explained variance in Figure 5. At the top, the row of face stimuli shows the colour code used in the mean ERP and modelled ERP plots, from blue (0% phase coherence) to red (100% phase coherence). The time course of the coefficient for the different model parameters is depicted in black, with the horizontal red line showing periods of statistically significant fitting (*p *< .01, not corrected for multiple comparisons). In the right column, purple lines show the mean coefficient across subjects and the thin black lines data from individual subjects. The beta coefficients are expressed in terms of signal change in *μ*V per unit of the predictor variable.

EEG results are illustrated in Figure 4. The mean ERP was modulated strongly by phase information starting at about 100 ms after stimulus onset and ending 200 ms later. The time-course of the explained variance (R^2^) reveals time windows in which the regression model provided a good fit to the data. For all subjects, explained variance started to rise just after 100 ms and peaked circa 150 ms, at about the same time the N170 peaked in the 100% phase coherence condition. There was no effect of the stimulus factor: presentation of face 1 or face 2 did not affect EEG amplitude. To confirm this result using a more traditional analysis, we compared systematically the mean single-trial EEG activity to face 1 and face 2, at all electrodes and time points, for each observer. Even with a very liberal univariate strategy (percentile bootstrap, 1000 resamples, *α *< .05, no correction for multiple comparisons), no significant effect was found in any observers. The same non-significant result was obtained when the analysis was performed on a more robust measure of central tendency, the 20% trimmed mean, rather than the mean. The same approach failed to reveal any significant difference in our two control experiments. Thus, the EEG activity in our experiment was not related to stimulus identity (i.e. face 1 vs. face 2, or texture 1 vs. texture 2).

**Figure 5:**
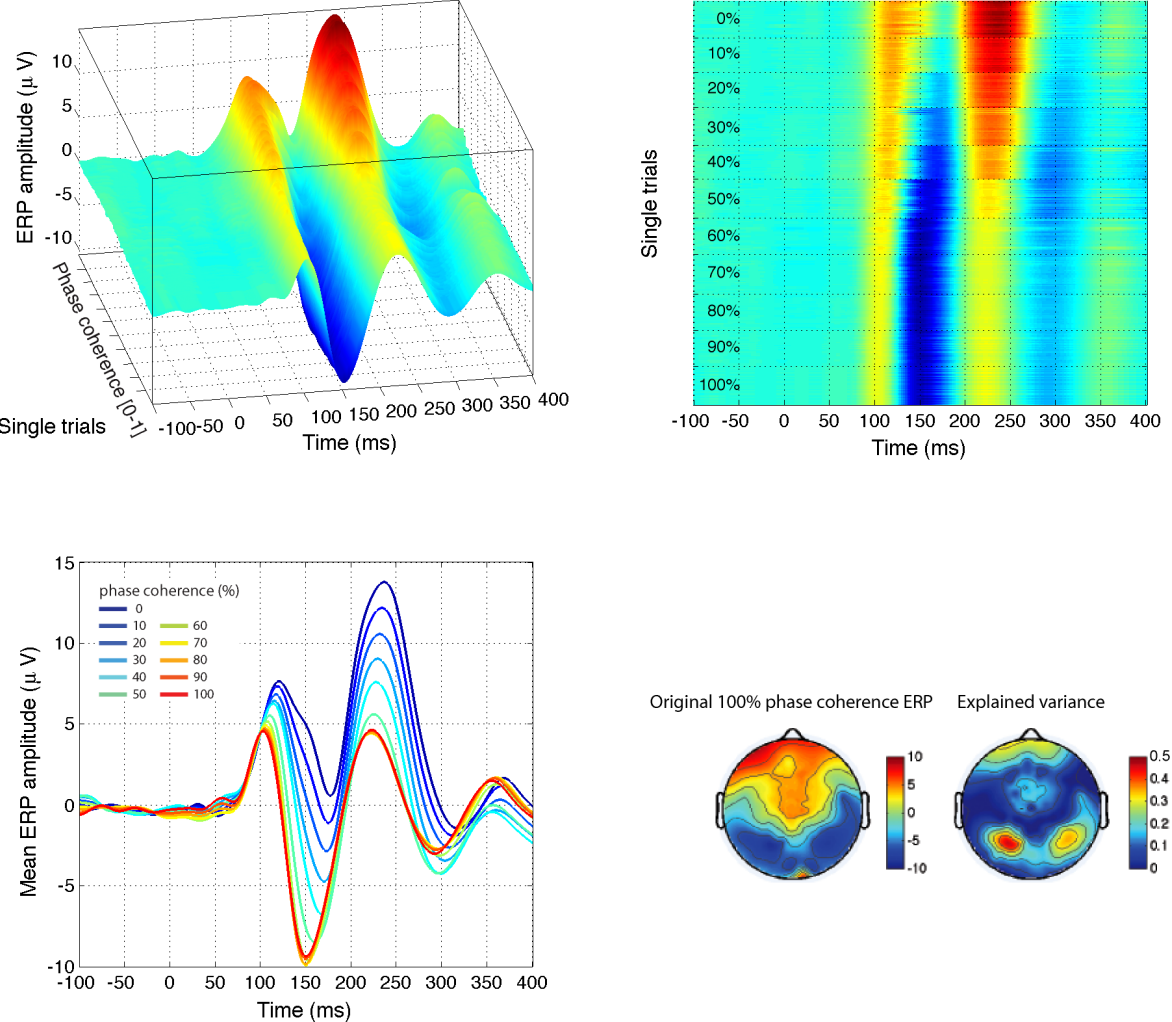
**Modelled data and topographical maps from session 1 of subject RXL in Experiment 1**. The upper part of the figure shows 3D (left) and 2D (right) representations of single trials, sorted by chronological order in which they were recorded during the experiment, independently for each bin of phase coherence. In the lower left corner, single-trial modelled data were averaged according to phase coherence level, and colour coded from blue (0%) to red (100%) following Figure 4 nomenclature. The data are from the electrode at which the maximum R^2 ^was obtained. The topographic maps show the interpolated ERP signal (left, in *μ*V) and explained variance (right, in %) at the latency of maximum R^2 ^(148 ms). The electrode showing the best fit is at the centre of the lower left red cluster in the explained variance map. For this subject, the electrode showing the maximum N170 was over the right hemisphere. However, at this electrode, the pattern of model fit was virtually indistinguishable from the one showed here.

The EEG signal was strongly modulated by phase coherence and kurtosis, but not skewness. Significant phase effects had a mean onset of 127 ms, kept increasing for about 30 ms and peaked at 160 ms after stimulus onset, at about the same time as the N170 (Table 1). More importantly, there was a significant phase × kurtosis interaction, starting at the transition between the P1 and the N170 components (150 ms), and reaching a maximum around the peak latency of the N170 (166 ms). There was no significant correlation between N170 latency and any of the model predictors. Phase, kurtosis, and phase × kurtosis effects where localized around the occipital-temporal electrodes with a maximum R^2^, specifically around electrodes PO7/8, P9/10, but also O1/2, with a right hemisphere preponderance (7/12 subjects). Table 1 reports maximum R^2 ^statistics in the three experiments. The interaction between phase and kurtosis can be visualized on the modelled data as a stronger phase modulation in the lower image kurtosis range. In other words, an increment in phase coherence had a stronger impact on single-trial amplitude when kurtosis was low compared to when kurtosis was high (Figure 5, top right). For the four subjects that were tested twice, the overall quality of the model and its time course were very consistent (overall correlations at the electrode with max R^2 ^for each subject: r = .92, r = .93, r = .97, r = .99, all p < .0001). Notably, only one subject, in one of his two sessions, did not show a significant phase × kurtosis interaction, suggesting that the phase and the phase × kurtosis interaction are very reliable (test-retest).

**Table 1:** Model fit results and 95% confidence intervals in the three experiments

		Experiment 1: Faces	Experiment 2: Pink Noise Textures	Experiment 3: Wavelet Textures
Max R^2^	Mean Amplitude	36.4 [29.2 44.6]	5.8 [3.6 8.3]	21.7 [12.4 30.4]
	Min Amplitude	19.9	2.8	5.5
	Max Amplitude	67.4	13.1	36.6
	Mean Latency	145 [142 148]	250 [228 272]	229 [180.8 296.6]
	
Phase	Onset Latency	127 [121 134]	NA	218 [161 263]
	Peak Latency	160 [154 168]	NA	228 [175 269]
	Onset-Peak Latency Difference	33 [26 42]	NA	10 [6 14]
	
Kurtosis	Onset Latency	127 [122 132]	NA	175 [163 199]
	Peak Latency	143 [137 148]	NA	204 [187 219]
	Onset-Peak Latency Difference	16 [13 19]	NA	29 [20 42]
	
Phase × Kurtosis	Onset Latency	150 [133 171]	NA	NA
	Peak Latency	166 [151 185]	NA	NA
	Onset-Peak Latency Difference	16 [12 20]	NA	NA

R^2 ^values are expressed as percentages. Latencies are expressed in ms. Regarding max R^2^, all the pairwise comparisons were significant (percentile bootstrap, 1000 resamples): Experiment 1 vs. Experiment 2, mean difference 30% [22 39], p = 0; Experiment 1 vs. Experiment 3, mean difference 15% [3 27], p = .009; Experiment 2 vs. Experiment 3, mean difference 16% [5 25], p = .002. Phase, Kurtosis, and Phase × Kurtosis interaction regressor time courses were analyzed at the electrode showing the max R^2 ^for each subject individually. Onset Latency refers to the earliest time at which a given model parameter contributed significantly to explaining the EEG data. NA stands for non-applicable, which corresponds to situations where only one subject showed a significant effect (Experiment 3) or the values are meaningless because of the poor model fit and the lack of significant effects (Experiment 2)

Across the four subjects, the mean value of the maximum R^2 ^was 36.2% (median = 35.7%; minimum = 19.8%, maximum = 67.3%).

**Figure 6:**
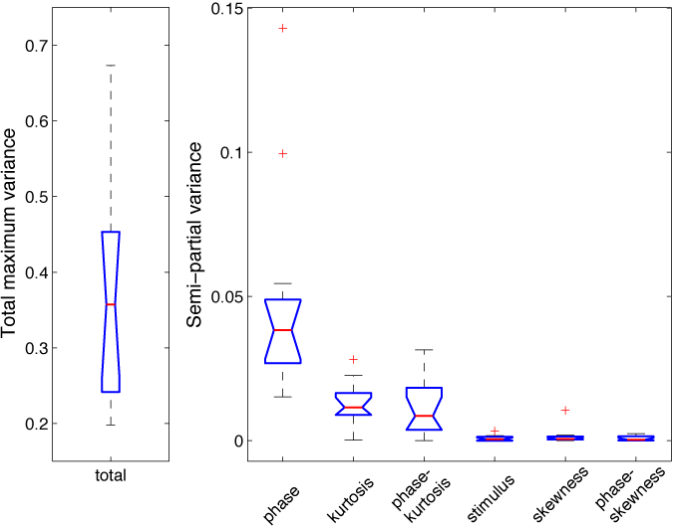
**Boxplots of total maximum explained variance and semi-partial variance in Experiment 1**. For each subplot, the red line indicates the median. The blue box extends from the upper to the lower quartile values. The whiskers show the most extreme points that are within 1.5 times the inter-quartile range. A red plus is an outlier. The notch in the blue box corresponds to a robust estimate of the median confidence interval. Non-overlapping notches indicate that medians differ with 95% confidence. Because of the scaling involved in the computation of the semi-partial variance, the sum of semi-partial variances across regressors is less than the total explained variance. The semi-partial variance calculation was performed at the electrode and time point of maximum R^2 ^peak for each subject.

The practical relevance of the different predictors was evaluated by the semi-partial variance across subjects. The semi-partial variance is a measure of the unique variance explained by one predictor after controlling for the effects of the other predictors, i.e. all other predictors were partialled out of the predictor of interest (for instance phase), but contrary to the partial variance, they were not partialled out from the dependent variable (our EEG response). The analysis of semi-partial variance revealed the significantly stronger impact of phase coherence on EEG single-trials, followed by kurtosis and the phase by kurtosis interaction, which did not differ from one another (Figure 6). Stimulus, skewness, and the phase by skewness interaction did not contribute significantly to explaining the EEG variance. This analysis is important, because it shows that both phase and kurtosis have unique effects, i.e. the effect of one cannot be explained simply by its linear association with the other (there was no significant quadratic relationship between phase and kurtosis).

**Figure 7:**
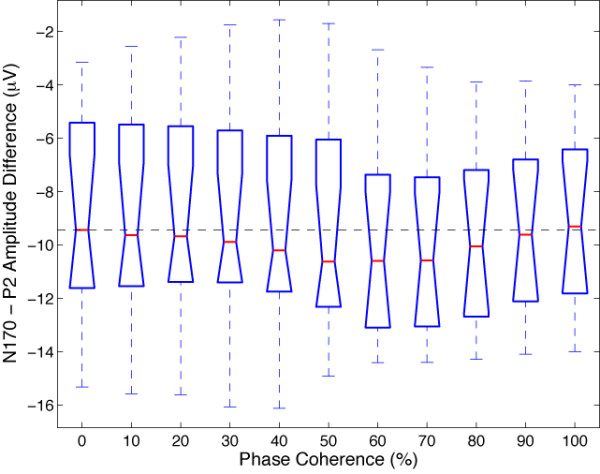
Boxplot of the peak-to-peak differences between the N170 and the P2 measured on modelled data at different levels of stimulus phase coherence for all subjects and sessions in Experiment 1.

Finally, in Figures 4 and 5, a larger P2 component (peaking between 200 and 250 ms) is apparent in response to noise patterns (0% phase coherence, in blue) compared to faces (100% face coherence, in red), a result that we have observed previously [28,37]. To determine if the difference in P2 amplitude was independent from the N170 amplitude, and might therefore reflect a mechanism of interest, a peak-to-peak analysis was carried out on the modelled data (Figure 7). Larger P2 amplitude in the 0% phase coherence condition, independently of the N170 amplitude, should lead to a decreasing peak-to-peak difference with increasing phase coherence. At 0% phase coherence, the peak-to-peak difference between the N170 and the P2 was -8.8 *μ*V with a 95% bootstrap confidence interval of [-10.7–6.9 *μ*V]. At 100% phase coherence, the peak-to-peak distance was very similar, with a mean of -9.2 *μ*V [-10.8–7.5 *μ*V]. The confidence interval for the difference between the two peak-to-peak differences was narrow and included zero [-0.2 0.9 *μ*V], thus failing to show a relatively larger P2 in response to noise textures. If anything, the peak-to-peak difference tended to increase from 0% up to about 70% of phase coherence, which goes against the idea that the difference between N170 and P2 amplitude might be reduced with increasing phase coherence. Therefore, it seems that the P2 difference is a simple carry-over of the N170 effect.

**Figure 8:**
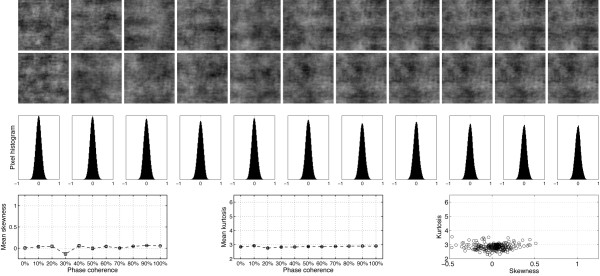
**Examples of stimuli used in Experiment 2**. The first two rows show the 22 stimuli presented to one observer during the first block of the experiment. The observer discriminated between the same two textures during the whole experiment. The noise level varied from 100% (left side; 0% phase coherence) to 0% (right side; 100% phase coherence). Histograms in the third row show the distribution of pixel contrasts averaged across all stimuli seen by this observer at each level of phase coherence. Note that, unlike the stimuli used in Experiment 1, the pixel histograms had a relatively constant Gaussian distribution across all levels of phase coherence. This constant histogram distribution is depicted in the last row, showing the mean skewness (left), and mean kurtosis (middle), as a function of phase coherence. The error bars correspond to 95% confidence interval computed using a bootstrap percentile technique (1000 resamples). In the bottom right end graph, kurtosis for each image (each circle) is expressed as a function of skewness. There was no relationship between the two statistical descriptors.

The results of Experiment 1 indicate that the event-related response to faces was not sensitive to face identity in our task. The main determinant of ERP variations was the structure of the stimuli, as captured by global phase coherence and kurtosis. To ensure that this was the case, a control experiment was conducted, in which the task was identical to the one employed in Experiment 1, but the target stimuli were pink noise textures created by randomizing completely the phase of the original face stimuli (Figure 8). This manipulation insured that the textures had the same amplitude spectrum as the faces used in Experiment 1, and that all textures had almost the same global statistics (Gaussian pixel histogram, constant kurtosis ~3) across all levels of phase coherence, while allowing subjects to perform the original task.

### Experiment 2: discrimination of two pink noise textures

**Figure 9:**
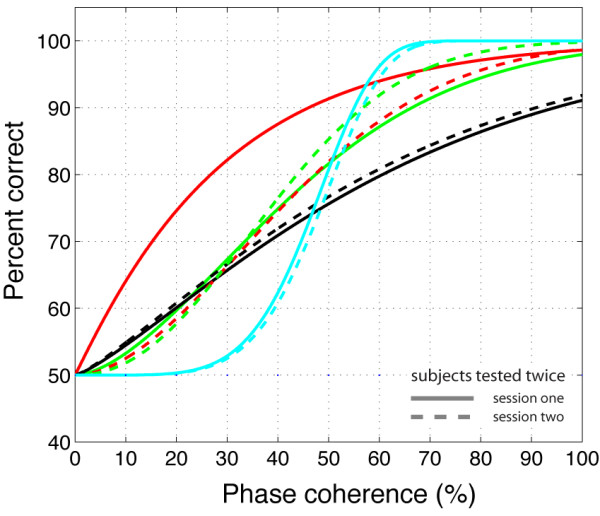
**Percent correct for individual subjects in Experiment 2**. The black lines show data from subject RXL. The other colours depict data from all other observers and sessions. Continuous lines indicate the first recording session, while dashed lines indicate the second recording session.

Although the textures used in Experiment 2 appear, at first glance, to be very similar, response accuracy in Experiment 2 was similar to the performance obtained with faces in Experiment 1 (Figure 9).

**Figure 10:**
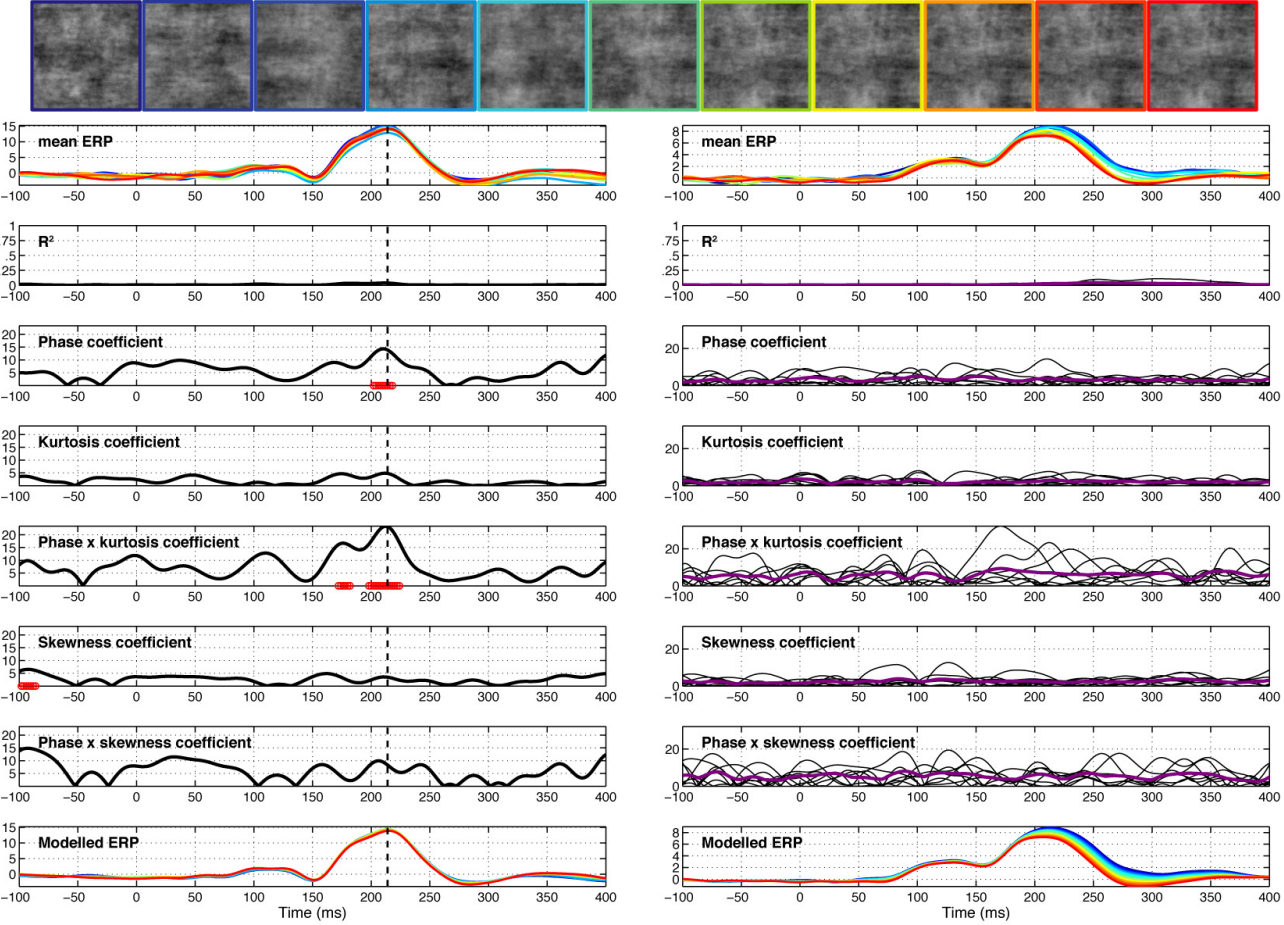
**Session 1 of subject RXL (left) and data averaged across subjects (right) in Experiment 2**. Figure caption details are otherwise identical to those of Figure 4.

**Figure 11:**
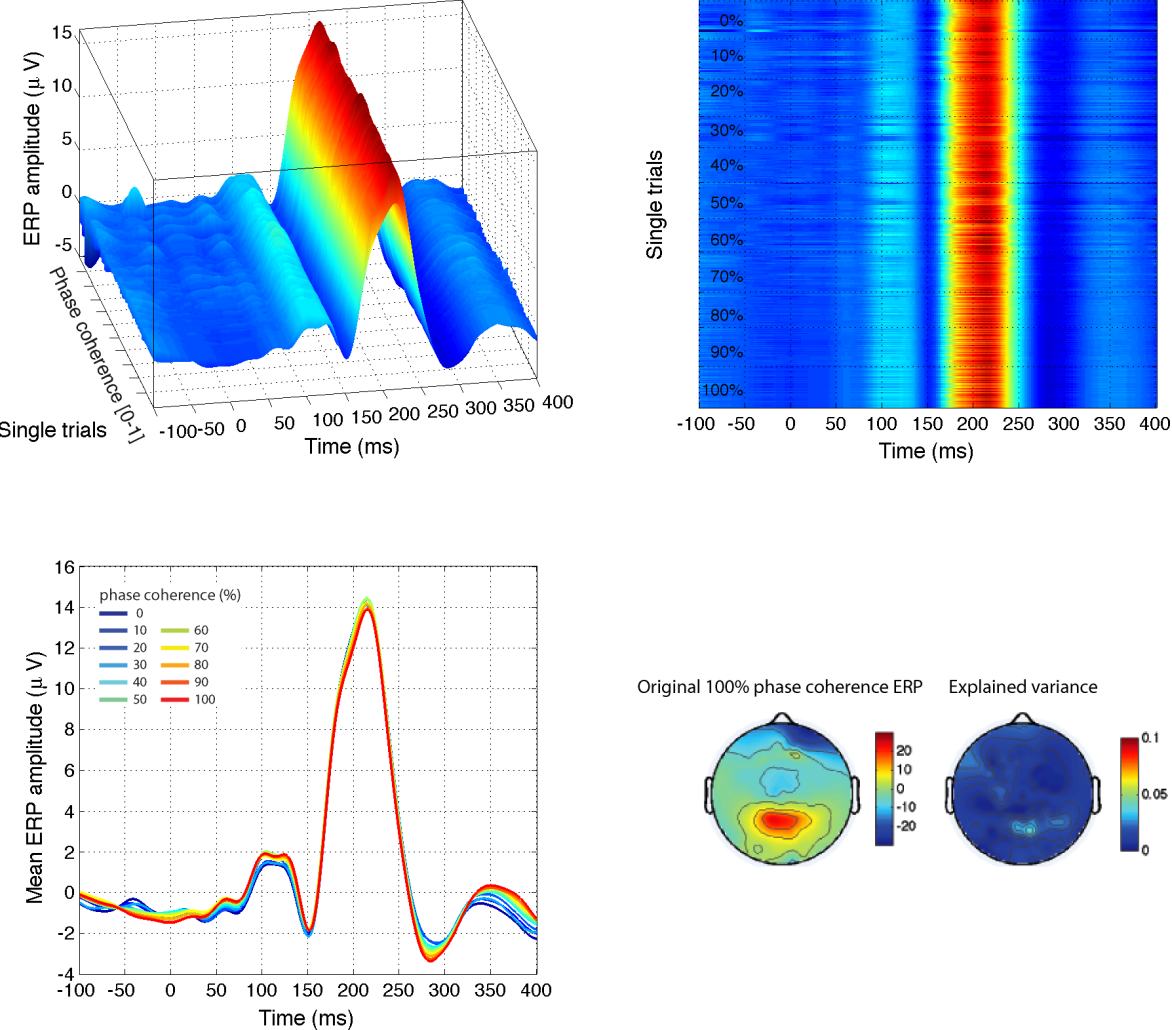
**Modelled data and topographical maps from session 1 of subject RXL in Experiment 2**. The topographic maps show data at the latency of maximum R^2 ^(214 ms). Figure caption details are otherwise identical to those of Figure 5.

**Figure 12:**
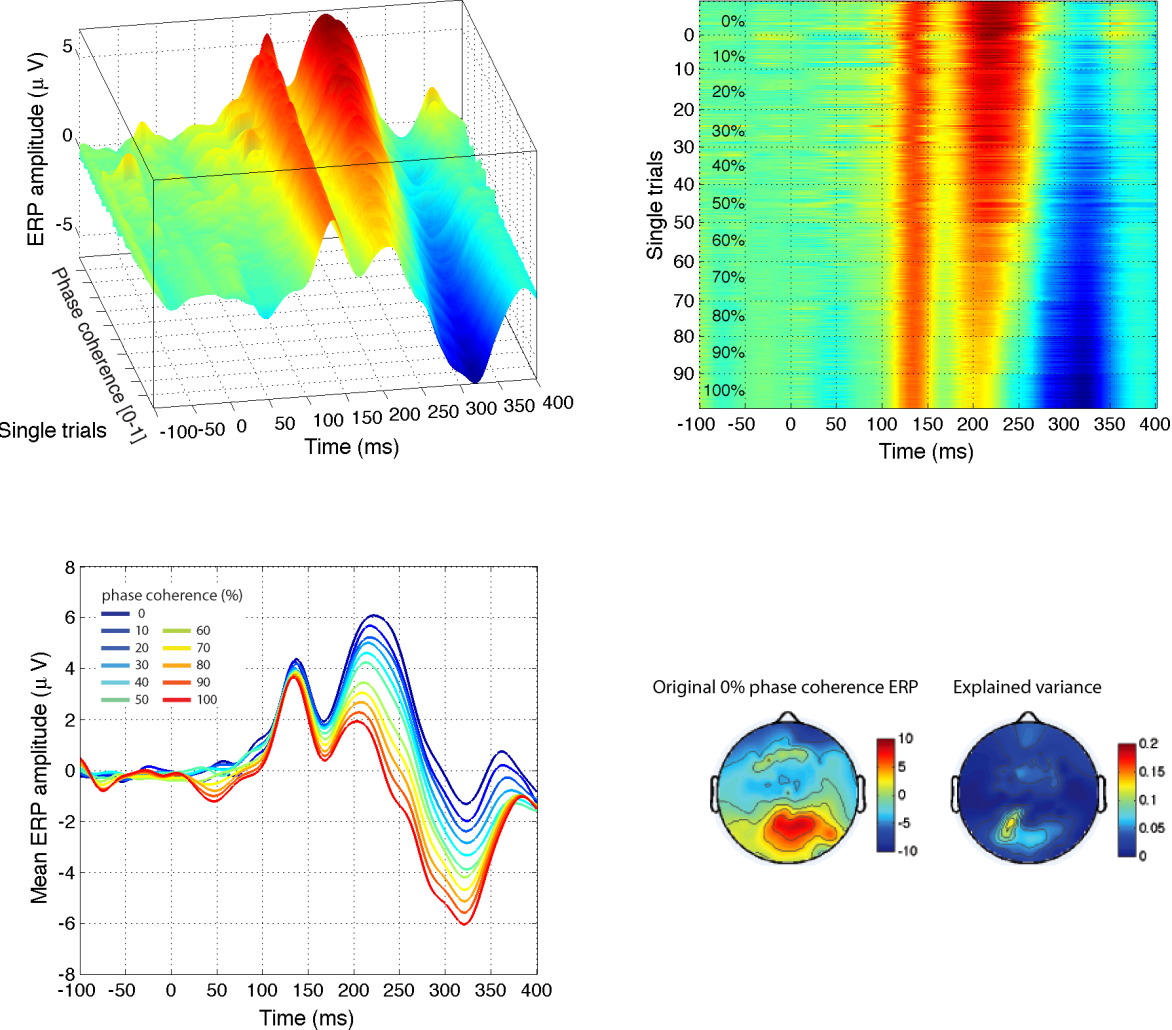
**Third EEG session from subject GAR in Experiment 2**. The topographic maps show data at the latency of maximum R^2 ^(242 ms). It appears that data from this subject were particularly sensitive to phase information, as shown by the gradient of amplitude responses in the modelled data. However, the largest amount of variance explained was relatively low (R^2 ^= 0.13). Moreover, during the time range 200–300 ms, when the model provided the best fit to the data, none of the regressors were associated significantly with the EEG signal. Only at about 350 ms, a few time points show a significant phase by kurtosis interaction, but in this latency range, the maximum R^2 ^= 0.05.

Although behavioural performance was very good in this task, pink noise textures failed to elicit the EEG pattern of sensitivity to phase noise evoked by faces in Experiment 1 (Figure 10). The global explained variance was extremely low (5.8%) compared to the high values previously obtained (36.4%), showing that our model failed to describe the data properly (Table 1). This result was true for all subjects, despite the fact that the pink noise textures elicited a very strong evoked response in the time range 150–250 ms post stimulus onset (Figures 10, 11 and 12). Even after extensive training with the same set of two stimuli there was no evidence of sensitivity to phase noise: Figure 12 shows data from a regular EEG session after 4408 trials, including previous EEG sessions and further behavioural practice between EEG recordings. Even in this case, the model provided a relatively poor fit of the data (max R^2 ^= 13.1%). In addition, that fit was delayed by at least 100 ms compared to the best fit obtained for faces in Experiment 1. Thus, it seems that the pattern of sensitivity to phase noise observed in response to faces is not related to task performance, i.e. subjects' capacity to discriminate between Stimulus A and Stimulus B, but might rather depend on the structure of the image.

### Experiment 3: discrimination between two wavelet noise textures

**Figure 13:**
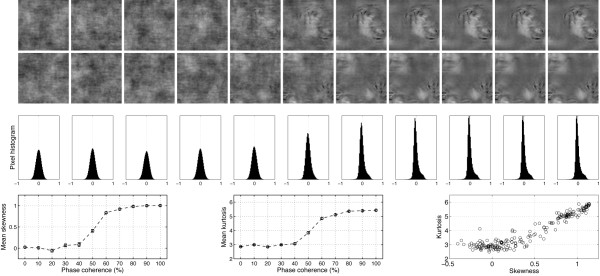
**Examples of stimuli used in Experiment 3**. The first two rows show the 22 stimuli presented to one observer during the first block of the experiment. The observer discriminated the same two wavelet textures during the whole experiment. The noise level varied from 100% (left side; 0% phase coherence) to 0% (right side; 100% phase coherence). Histograms in the third row show the distribution of pixel contrasts averaged across the entire set of stimuli seen by this observer at each level of phase coherence, from 0% (left) to 100% (right). Similarly to what was observed for faces in Experiment 1, and contrary to the pink noise textures used in Experiment 2, the pixel histograms showed a non-linear transition from a Gaussian distribution to a skewed and kurtotic distribution with increasing levels of phase coherence. This progression is depicted in the last row, showing the mean skewness (left), and mean kurtosis (middle), as a function of phase coherence. The error bars correspond to 95% confidence interval computed using a bootstrap percentile technique (1000 resamples). In the bottom right end graph, kurtosis for each image (each circle) is expressed as a function of skewness. Similarly to face stimuli, the two statistical descriptors are non-linearly related to one another.

**Figure 14:**
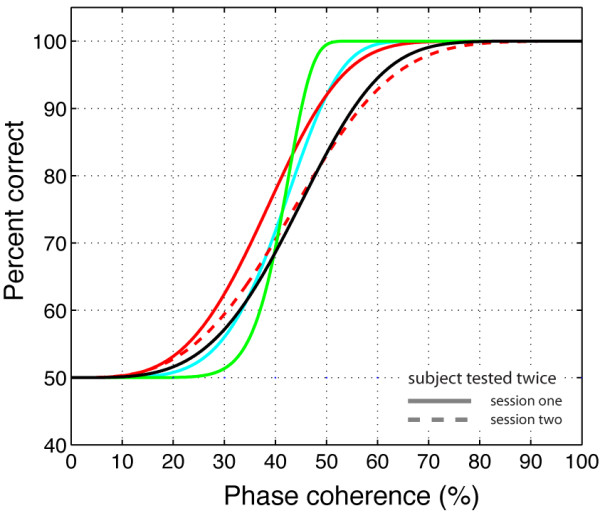
**Percent correct for individual subjects in Experiment 3**. The black lines show data from subject RXL. The other colours depict data from all other observers and sessions. Continuous lines indicate the first recording session, while dashed lines indicate the second recording session.

Finally, we performed a last control experiment to determine whether textures with the same higher-order image statistics as faces were sufficient to elicit the pattern of sensitivity to phase noise observed in Experiment 1. Unlike the pink noise textures used in Experiment 2, the kurtotic textures created for Experiment 3 did contain local elements (edges and 'blobs') that were similar to those seen in faces (Figure 13). With these textures, behavioural performance was very good for all subjects (Figure 14).

**Figure 15:**
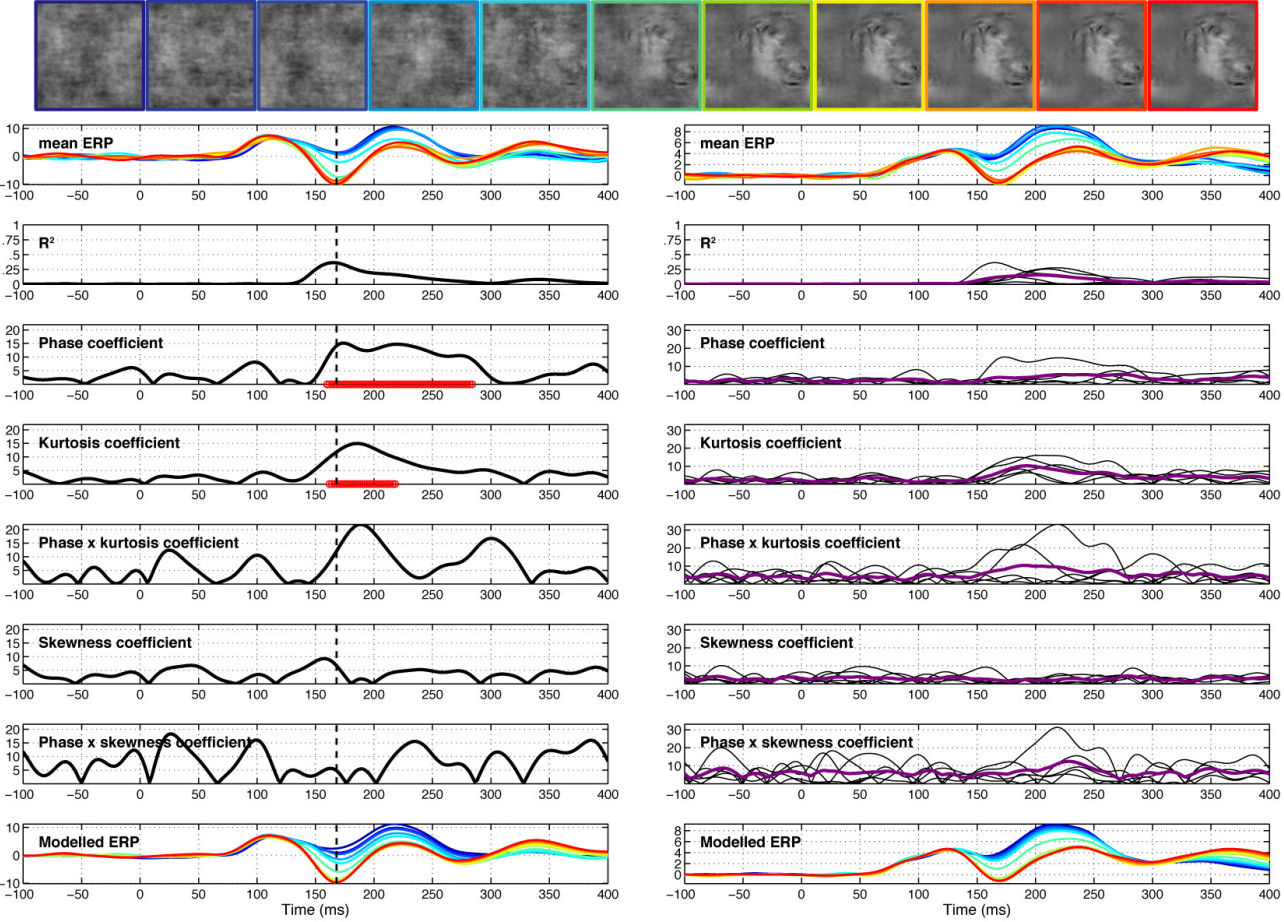
**Session 1 of subject RXL (left) and data averaged across subjects (right) in Experiment 3**. Figure caption details are otherwise identical to those of Figure 4.

**Figure 16:**
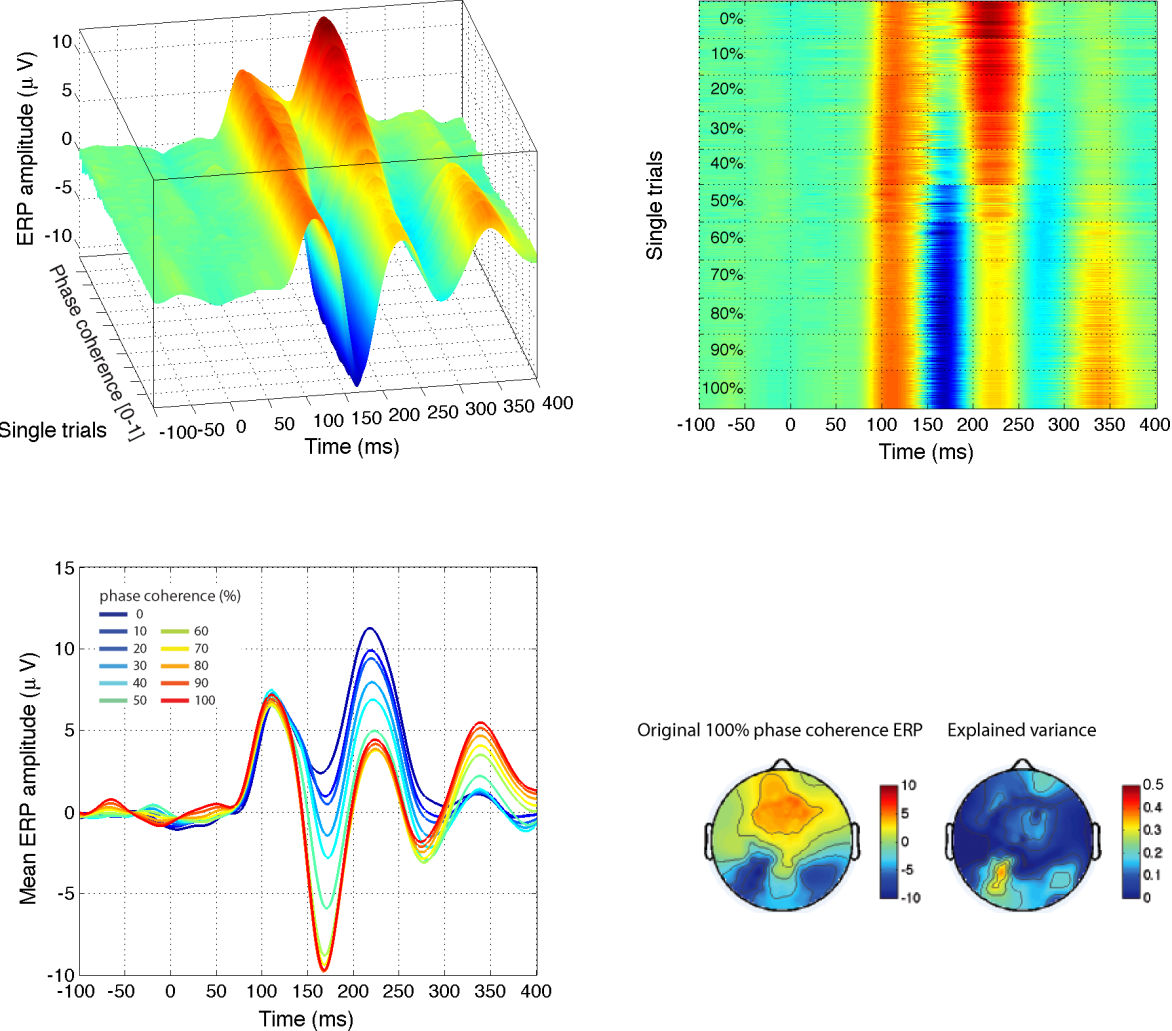
**Modelled data and topographical maps from session 1 of subject RXL in Experiment 3**. The topographic maps show data at the latency of maximum R^2 ^(168 ms). This subject showed the earliest and the strongest sensitivity to phase noise. However, sensitivity to phase noise only starts to emerge at about 150 ms after stimulus onset, compared to about 120 ms in Experiment 1. Figure caption details are otherwise identical to those of Figure 5.

Importantly, EEG activity showed a clear N170 effect, with a much stronger signal for 100% phase coherence stimuli compared to 0% phase coherence stimuli between 150 and 200 ms after stimulus onset. This response pattern contrasted sharply with the response generated by pink noise textures, showing no N170 effect, and a very poor model fit overall. This activity was modulated by phase coherence in three subjects out of five (Figures 15 and 16). However, despite some similarities, the EEG response to wavelet textures did not match completely the response triggered by faces. Particularly, the model provided a lower and delayed fit of the data compared to Experiment 1 (Table 1). Furthermore, the phase × kurtosis interaction was significant in only one of the subjects (onset = 163 ms, peak latency = 205 ms). Finally, an analysis of the semi-partial variance (similar to the one presented in Figure 5) revealed a very weak unique contribution of phase (min = 0, max = 0.009) compared to the one observed for faces (min = 0.015, max = 0.143), and no significant difference among the unique contributions of the different regressors (*F*_(5,20) _= 2.1, *P *= 0.4). Although limited by the small number of subjects tested in our second control experiment, these results suggest that higher-order image statistics of natural objects, in this case faces, are necessary but not sufficient to explain the pattern of EEG sensitivity to phase information observed for faces. A direct comparison between the sensitivity to phase noise of faces and other stimulus categories, like wavelet textures, will require further testing using a parametric and categorical mixed design.

## Discussion

Our previous work showed that early evoked brain activity to faces and objects, starting at about 130–150 ms after stimulus onset, does not reflect differences in stimulus amplitude spectra, but rather is mainly driven by spatial phase information [28,36,37]. The current results reveal for the first time the time course of the brain sensitivity to global visual phase information. In other words, we determined how phase information shapes early brain responses to complex objects like faces. Our results show that the visual system sensitivity to global phase information emerges progressively in a short time window between the P1 and the N170 components. This sensitivity to phase noise starts roughly at about 120–130 ms after stimulus onset and continues for another 25–40 ms, as indicated by the time course of model R^2 ^and the regression fit (Figure 4). During this delay, single-trial activity is not only sensitive to phase information, but also to kurtosis, and the two global image descriptors interact significantly with each other (Figures 4 and 5).

Our linear phase manipulation introduced non-linear monotonic changes in higher-order image statistics, as revealed by kurtosis measurements [46]. Kurtosis is a good measure of image sparseness [50,52,61]. The monotonic and non-linear increase in kurtosis, from 0% to 100% phase coherence, thus most probably corresponds to the build up of local elements like edges and contours, that in turn are formed by local phase alignment across different spatial frequency bands [38,40,50]. The time course of our model fit might thus reveal the extraction of global image structure, and not only sensitivity to phase information.

We note that our kurtosis measurements were made directly on the pixel contrasts. Thomson [50] warned about measuring kurtosis from non-whitened images because coloured noise (in our case pink noise corresponding to the 0% phase coherence condition) contains pixel-wise correlations that might inflate kurtosis artificially, rendering it a non-interesting measure to detect sparseness in images. However, we were interested in the relative differences in kurtosis across image types, and, more importantly, our pink noise textures were appropriately centred around 3, the value expected from white noise distribution, not contaminated by 1/f amplitude spectrum information.

Two control experiments (Experiments 2 and 3) showed that higher-order statistics of faces are necessary but not sufficient to obtain the sensitivity to phase noise observed in response to faces (Experiment 1). First, although in Experiment 2 subjects' could discriminate two pink noise textures presented in the same conditions as faces in Experiment 1, the regression model failed to fit the data. Second, wavelet textures matched for both global and some of the local image face statistics did trigger a face-like EEG pattern, but in this case the model provided a poorer overall fit to the data, a delayed timing in the fit, and an absence of phase × kurtosis interaction. This result points to particular local phase arrangements as being responsible for the model fit observed for faces. This is not a trivial point, because it would be conceivable to observe a time-course of phase noise sensitivity for control textures that would be similar to the one observed for faces. This possibility stems from the fact that the linear regression fit used to measure sensitivity to phase noise is independent of the global shape of the ERP, i.e. its mean. Our second control experiment also raises the question of how far we can go into matching image statistics without simply reproducing the stimulus. From our results, it remains unclear how the EEG to faces and matched wavelet textures precisely compare within subjects, and this will require further investigation using a paradigm in which the two types of stimuli, as well as control object categories, are tested in the same recording session.

Parametric stimulus phase manipulations and, more generally, parametric noise manipulations have been used in the literature to investigate the spatial and temporal hierarchical encoding of visual information. In the spatial domain, fMRI has lead to the discovery of various noise tuning functions in different brain areas of human and non-human primates. The general conclusion from those studies is that there is an increasing sensitivity to noise along the ventral pathway, from V1, where for instance the signal evoked by natural images does not differ from the one evoked by pink noise [9,62], but see [63], to higher-level object processing areas, where noise sensitivity tends to be the strongest [7,9,63,64]. The strongest noise sensitivity in higher levels of the visual hierarchy as observed in fMRI, together with EEG and MEG source analyses of evoked activity in the time range of the N/M170 [16,65,66], suggests that the sensitivity to phase noise we recorded in response to faces corresponds to the activity of face or object processing areas integrating information about the global structure of the stimulus. Future studies should investigate the cortical network involved in the effects reported here, as well as the nature of these effects, essentially feedforward or reflecting the integration of information from other structures [67,68].

In the temporal domain, EEG and MEG studies have reported results compatible with our findings. For instance, additive noise has been used to dissociate the stimulus sensitivity of early evoked responses. In EEG, it has been demonstrated that there is a linear inverse relationship between the amount of white noise added to face stimuli and the N170 amplitude. In contrast, the earlier P1 component is not affected by this noise manipulation [29]. In MEG, another type of dissociation has been reported between the M1 and the M170, which are to some extent the magnetic counterparts of the P1 and N170 ERP components [66]. In a parametric design in which faces were masked by narrow band-pass filtered noise, Tanskanen et al. found that the M170 amplitude was modulated by noise spatial frequencies in a very similar manner to recognition performance [32]. When noise patterns were presented in the absence of face stimuli, the spatial frequency noise sensitivity of the M170 disappeared, whereas the earlier M1 component showed frequency tuning similar to the one triggered by face + noise stimuli. It thus seems clear from these two studies that the N170 reflects, at least in part, the activity of a mechanism that begins to respond during the N170 time window and which was not active during earlier time frames.

In our study, we made the explicit assumption that this mechanism might be related to global phase processing. We also provide for the first time a detailed timing of sensitivity to phase noise, using a single-trial model incorporating only parameters related to the global image statistics. Our approach did not depend on the identification of traditional ERP components, and therefore allowed us to track sensitivity to phase noise without limiting our analyses to ERP peaks. This aspect of the analyses is important, because we found that information extraction starts at the *transition *between the P1 and the N170. This result is in keeping with a series of recent studies, relying on component-free single-trial models, showing onsets of task related information accrual just before the N170, but after the P1 [14,30,31,69].

## Conclusion

Our approach was not to map the relationship between behaviour and EEG activity, but rather to focus on global image properties and how they shape early EEG activity. This is why we kept the task simple and constant. This approach is legitimate because early brain activity evoked by some categories of complex objects, like faces and words, is hardly modulated by task factors ([27,28], but see some recent advances in [14,69]), and because there is still much to learn about the relationship between image statistics and brain activity [61]. Furthermore, our approach provides a potentially fruitful departure from frameworks that make assumptions about stimulus space and the nature of relevant 'features'. Like natural scenes, faces can receive a statistical description rather than a category label. For instance, contrasting faces and houses can tell us a great deal about when and where in the brain these two kinds of stimuli are differentially represented. However, other properties co-vary with these semantic categories, and it remains unclear precisely what type of information is extracted when a categorical difference is observed. Using parametric designs can circumvent this limitation. In that framework, phase manipulations constitute one way to explore face and object processing by creating an information space. This approach can be understood as an extension of classic categorical designs, in which regular stimuli are contrasted with noise (i.e. the two extreme points of the continuum). Finally, our approach can be applied to a large range of problems that researchers in behavioural neuroscience usually address using categorical designs.

## Authors' contributions

GAR, PJB, ABS designed the study. GAR collected the data, conducted the analyses and wrote the manuscript. CRP helped analyse the data. CRP and PJB helped revise the manuscript. All authors read and approved the final manuscript.
